# A pure de novo 16p13.3 duplication and amplification in a patient with femoral hypoplasia, psychomotor retardation, heart defect, and facial dysmorphism—a case report and literature review of the partial 16p13.3 trisomy syndrome

**DOI:** 10.1007/s13353-022-00743-7

**Published:** 2022-12-31

**Authors:** Magdalena Socha, Anna Szoszkiewicz, Dorota Simon, Aleksander Jamsheer

**Affiliations:** 1 Department of Medical Genetics, Poznan University of Medical Sciences, Rokietnicka 8, 60-806 Poznań, Poland; 2 Centers for Medical Genetics GENESIS, Dąbrowskiego 77A, 60-529 Poznań, Poland

**Keywords:** 16p13.3 duplication, Psychomotor retardation, Femoral hypoplasia, Partial 16p trisomy syndrome

## Abstract

Partial 16p trisomy syndrome is a rare disorder typically characterized by psychomotor retardation, prenatal and postnatal growth deficiency, cleft palate, and facial dysmorphism, with some patients also presenting with heart defects and urogenital anomalies. Pure 16p13.3 duplications usually occur de novo, while those duplications that associate with partial monosomy result rather from parental chromosomal translocations. Due to the large size of the aberrations, the majority of patients are identified by standard chromosome analysis. In all published cases, the minimal-causative duplicated region encompasses the *CREBBP* gene. Here, we report on the patient presenting with psychomotor retardation, femoral hypoplasia, and some features of the partial 16p trisomy syndrome, who carries a complex de novo terminal 16p13.3 microduplication with an overlapping region of amplification without translocation or associated monosomy. In contrast to the previously reported cases, the duplicated region of the patient does not involve *CREBBP* and other neighboring genes; still, the observed pattern of dysmorphic features of the index is characteristic of the described syndrome. Based on the animal studies and other published cases, we discuss the possible role of the *PDK1* and *IGFALS* genes in the development of limb anomalies, while *IFT140* could contribute both to the observed femoral phenotype and heart abnormalities in the patient. To the best of our knowledge, we present a proband harboring the smallest terminal 16p13.3 duplication of the size below 3 Mb. Therefore, our proband with her detailed phenotypic description may be helpful for clinicians who consult patients with this syndrome.

**Supplementary Information:**

The online version contains supplementary material available at 10.1007/s13353-022-00743-7.

## Introduction

The 16p13.3 duplication is a rare congenital multiple anomaly syndrome clinically characterized by psychomotor retardation, including delayed speech development, intellectual disability, behavioral disturbance encompassing autism spectrum disorders, and/or attention-deficit hyperactivity disorder. Additionally, congenital heart defects, skeletal abnormalities (i.e., hip dislocation, camptodactyly, digital shortening), and facial dysmorphism are noted (Babameto-Laku et al. 2012; Chen et al. 2012; Demeer et al. 2013; Fontes et al. 2016; Li et al. 2013; Mohamed et al. 2015; de Ravel et al. 2005; Thienpont et al. 2010). The clinical phenotype is highly variable among affected individuals, depending on the size of the duplicated region and its gene content. Although 16p13.3 duplications significantly differ in size and genomic location, most of the patients presenting with the typical features of the syndrome carry duplications involving the *CREBBP* gene (Demeer et al. 2013). In addition, the syndrome usually occurs due to de novo mutations, while less frequently is inherited from a mildly affected parent or results from a balanced parental chromosomal aberration encompassing 16p13 region.

Hypoplasia of the femoral bones is a rare congenital long bone anomaly with only a few known pathogenic genetic variants underlying the disorder. Recently, several causative copy number variations (CNVs) have been identified in patients with either isolated or syndromic bilateral femoral hypoplasia, including 10q24.32 or 2q37.3 microduplications, respectively (Socha et al. 2021; Spielmann et al. 2016). Interestingly, some individuals with 16p13.3 duplication described in the literature also presented with rhizomelic length reduction of limbs due to femoral bone shortening. In this report, we describe the first patient with 16p13.3 duplication associated with the putative triplication (or more significant amplification) of a small 16p13.3 segment. The index carries a pure complex 16p13.3 chromosomal microaberration and displays a rare phenotype of femoral bone hypoplasia and bowing, as well as fractures of the femoral bones. Here, we also provide the literature review and discuss possible genotype–phenotype correlations for the 16p13.3 terminal duplication syndrome.

## Materials and methods

### Patient’s material

All patients agreed to participate in this study. Written informed consent was obtained from all individuals or their legal guardians prior to genetic testing. This study was approved by the Institutional Review Board of the Poznan University of Medical Sciences ethics committee. Genomic DNA was extracted from patients’ peripheral blood leukocytes by standard procedures.

### Cytogenetic testing

Prenatal standard GTG banding at resolution 400 bands per haploid genome was performed following an amniocentesis. For determining karyotype, 20 metaphases were analyzed and described according to the International System for Human Cytogenetic Nomenclature (McGowan-Jordan et al. 2020).

Standard GTG banding at a resolution of 550 bands per haploid genome was performed on the peripheral blood cells of the index patient. Karyotype determining was as described above.

### FISH

Fluorescent in situ hybridization (FISH) was performed on a suspension of metaphase cells in a fixer 3:1 mixture of methanol and acetic acid. A standard culture of lymphocytes derived from the proband’s peripheral blood was performed in the RPMI 1640 medium for 72 h at 37 °C; next, colcemid was added to the culture medium in order to arrest the cells in metaphase. Dual-color FISH was performed using the Kreatech™ Sub-Telomere FISH Probes KBI-40228-R 16pter red and KBI-40229-G 16qter green (Kreatech Diagnostics, Amsterdam, the Netherlands) according to the manufacturer’s shortened instructions; i.e., heat denaturation of the DNA on a slide and adding the probes were performed on a heating block. Fluorescent Nikon Eclipse E400 microscope with triple band-pass filters (DAPI/FITC/Rhodamine) was used to visualize the chromosomes, and 20 metaphases were scored.

### Array CGH

Array comparative genomic hybridization (array CGH) was performed using 1 M oligonucleotide array (Agilent™, Agilent Technologies, Santa Clara, CA) following the producer’s protocol. Feature extraction and analysis were done using Agilent CytoGenomics 5.0.2.5 software (Agilent™, Agilent Technologies, Santa Clara, CA). Analysis settings are as follows: aberration algorithm: ADM-2; threshold: 6.0; window size: 0.2 Mb; filter: 3 probes, log2ratio = 0.29. Copy number variants (CNVs) were determined using thresholds of 0.4 for gains and − 0.4 for losses. Commercial Agilent™ Euro Female reference genomic DNA was used for hybridization.

#### Databases and in silico analysis

For the CNV assessment, the following databases were used: DECIPHER (https://decipher.sanger.ac.uk/), ClinVar (http://www.ncbi.nlm.nih.gov/clinvar/), DGV (http://dgv.tcag.ca/dgv/app/home), and gnomAD SVs (v2.1) (https://gnomad.broadinstitute.org/).

### qPCR and co-segregation analysis

Quantitative real-time polymerase chain reaction (qPCR) was utilized to validate the identified CNVs and perform co-segregation analysis in the proband’s parents. Primers used for the qPCR targeted the duplication region (3 pairs), amplification region (2 pairs), deletion region (1 pair), and a normal region in the vicinity of the duplication located downstream at chromosome 16p13 (1 pair). We followed a previously described protocol (Sowińska-Seidler et al. 2015) and ran samples in triplicate on the ViiA™ 7 Real-Time thermal cycler (Applied Biosystems). For normalization, we used the albumin gene (*ALB*). To test the accuracy of sample preparation, we included a primer pair targeting the factor VIII gene (*F8*), which is located at chromosome X. The copy number analysis was performed using the comparative 2^−ΔΔCT^ method, with the use of healthy individual’s DNA as a calibrator. See Online Resource 1 for primer sequences.

## Results

### Clinical report

**Fig. 1:**
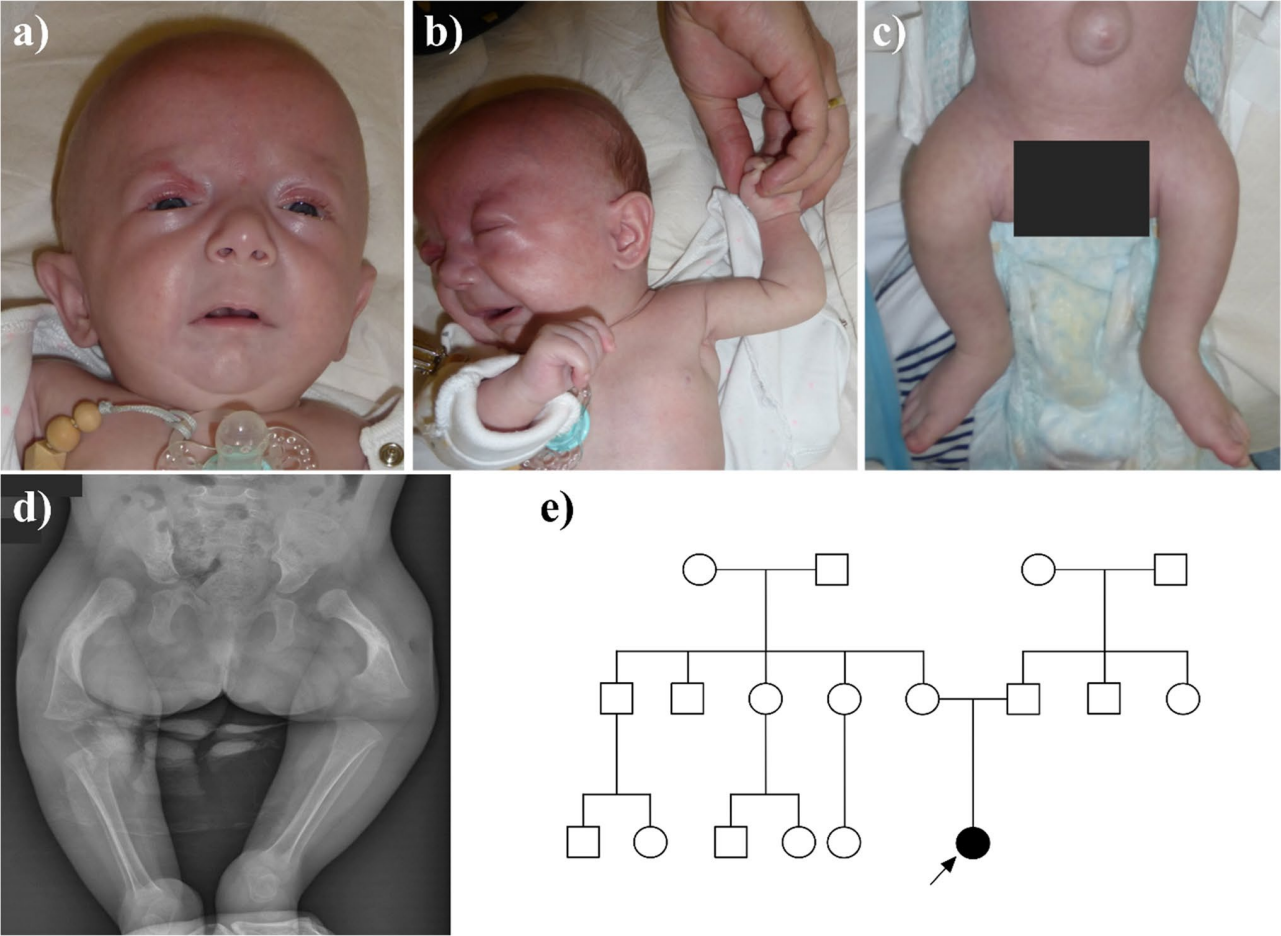
Clinical and radiological picture of the index patient and the pedigree. **a** Facial view of the proband showing round face, full cheeks, micro- and retrognathia, hypertelorism, depressed nasal bridge, bulbous nasal tips, low-set ears, shallow orbits, and flattening of the philtrum with hypoplastic fossa philtrum. **b** Mildly shortened humeral segments and markedly shortened femoral segments, as well as a marked umbilical hernia (**c**). **d** A radiograph of the proband at the age of 10 months showing shortened and bowed femora with increased ossification of the femoral shafts. **e** Pedigree of the family, the black arrow points to the proband, who is the only affected individual

A 2.5-year-old female patient was born from the 1st pregnancy to a healthy, nonconsanguineous couple in the 32nd week of gestation by cesarean section due to the premature rupture of membranes and pelvic position of the fetus. Prenatal ultrasound scan at 17 weeks + 3 days of gestation revealed bilateral shortening of the femoral bones, single umbilical artery, ventricular septal defect (VSD), and abnormal facial profile. Amniocentesis followed by a prenatal GTG banding showed a normal female karyotype. After birth, the proband’s weight was 1300 g (10th–25th percentile), length 38 cm (10th–25th percentile), head circumference 30 cm (50th–75th percentile), and the Apgar score was 8 and 8 at 1ʹ and 10ʹ, respectively. Clinical examination revealed a midline cleft of the soft and hard palate; marked umbilical hernia; and malformed, bowed, and markedly shortened femoral segments, as well as less prominently shortened humeral segments, limitation of abduction in the hip joints, partial syndactyly of toes 2–3 in the right foot, and conductive hypoacusis, probably secondary to the cleft palate. Due to the shortening of the proximal limb segments, the crown-rump length (CRL) of 29 cm was measured. VSD detected prenatally and after birth was hemodynamically insignificant. In control echocardiography at the age of 1 month, only tricuspid valve insufficiency of I/II degree was noted (TI I/II°). Facial dysmorphism included a round face, full cheeks, micro- and retrognathia, hypertelorism, depressed nasal bridge, bulbous nasal tip, low-set ears, shallow orbits, and flattening of the philtrum with marked hypoplasia of the fossa philtrum (Fig. 1).

Fontanelle ultrasonography revealed a few subependymal cysts, which showed regression in the control study. The abdominal ultrasound scan was normal. Further clinical and radiographic examination revealed a non-displaced fracture of the right femur and bilateral skin dimples in the 1/3 length of the lateral sides of both thighs. Additionally, during an orthopedic and X-ray examination at the age of 5 weeks, a right-sided multi-fragment femoral shaft fracture in the status of fusion and sclerotization was diagnosed, as well as movement restriction of the knee joints was noted.

A deterioration of the cardiopulmonary condition occurred twice, shortly after birth and again on the 15th day. Therefore, the patient required respiratory support with nCPAP for a period of 24 h, followed by passive oxygen therapy. During the second aforementioned episode, tachycardia, pale skin with cutis marmorata, and respiratory acidosis were noted. Failure to thrive and frequent regurgitation were observed. The index underwent a surgical correction of the cleft lip and palate at the age of 11 months. Psychomotor development was delayed, with independent sitting achieved at 12 months, standing up with assistance at 21 months, and walking with assistance at 23 months of age. At 30 months of age, the patient is able to stand up, however cannot walk independently, weighs 8 kg, and has a height of 75 cm, which is below the 3rd percentile for both measurements. The first word was spoken at 11 months of age, albeit the speech has not developed further, most probably due to the ankyloglossia and complete fusion of the tongue on its entire length to the floor of the mouth. At the age of 2.5 years, micro- and retrognathia and flattening of the philtrum are still marked. There is a bilateral hyperopia (+ 5.0 Dsph), and the patient speaks only 4 words, although the hearing assessed in ABR control investigations is normal. The femoral bones are shortened, bowed, and grow very slowly in length, resulting in bilateral rhizomelic lower limb shortening. There were no further femoral fractures; however, a dislocation of the left hip with aplasia of the left acetabulum as well as hypoplasia of the right acetabulum was diagnosed.

### Cytogenetic testing

Following abnormal prenatal ultrasonography, amniocentesis was performed, and the fetal karyotype was determined as normal female karyotype: 46,XX.

**Fig. 2:**
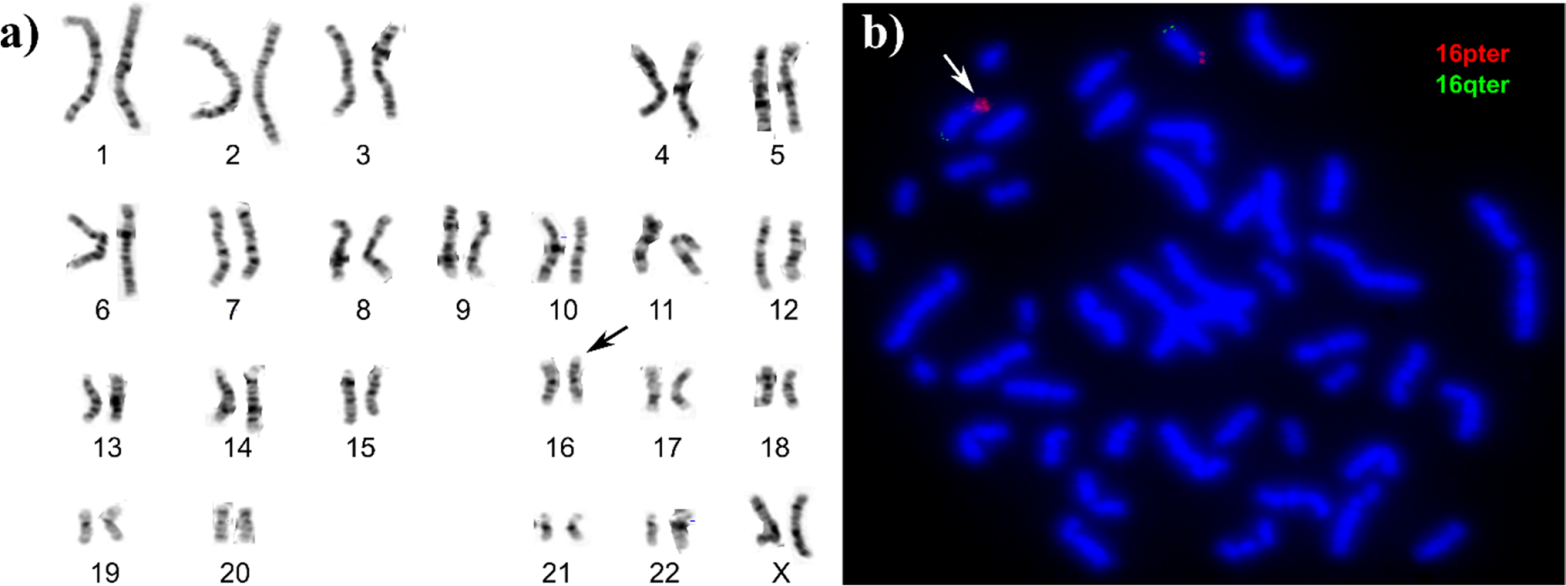
Results of postnatal karyotype and FISH. **a** Standard chromosome analysis of the index patient revealed abnormal karyotype: 46,XX,dup(16)(pter- > p13.3::p13.3- > qter); the black arrow points to the additional material on the short arm of chromosome 16. **b** FISH performed using Kreatech Sub-Telomere Probes for 16pter and 16qter demonstrated the normal two signals of subtelomere 16q (spectrum green), one normal signal of subtelomere 16p (spectrum red), and an abnormal signal of the other subtelomere 16p, indicating an additional genomic material originating from the short arm subtelomeric region of chromosome 16 (the white arrow points to the aberrant chromosome)

Due to multiple inborn anomalies observed in the index patient, chromosome analysis was repeated and revealed an unbalanced female karyotype: 46,XX,dup(16)(pter- > p13.3::p13.3- > qter). A terminal duplication of a short arm of chromosome 16 was detected, with the breakpoint located at 16p13.3 (Fig. 2a).

### FISH

Fluorescence in situ hybridization (FISH) using probes specific to the chromosome 16 subtelomeres demonstrated that the additional genomic material is located terminally on the short arm of one chromosome 16, in the same cytogenetic region in which the described duplication and amplification were detected. Neither other chromosome carried a signal for the 16pter probe nor was a deletion detected (Fig. 2b).

### Array CGH and validation

**Fig. 3:**
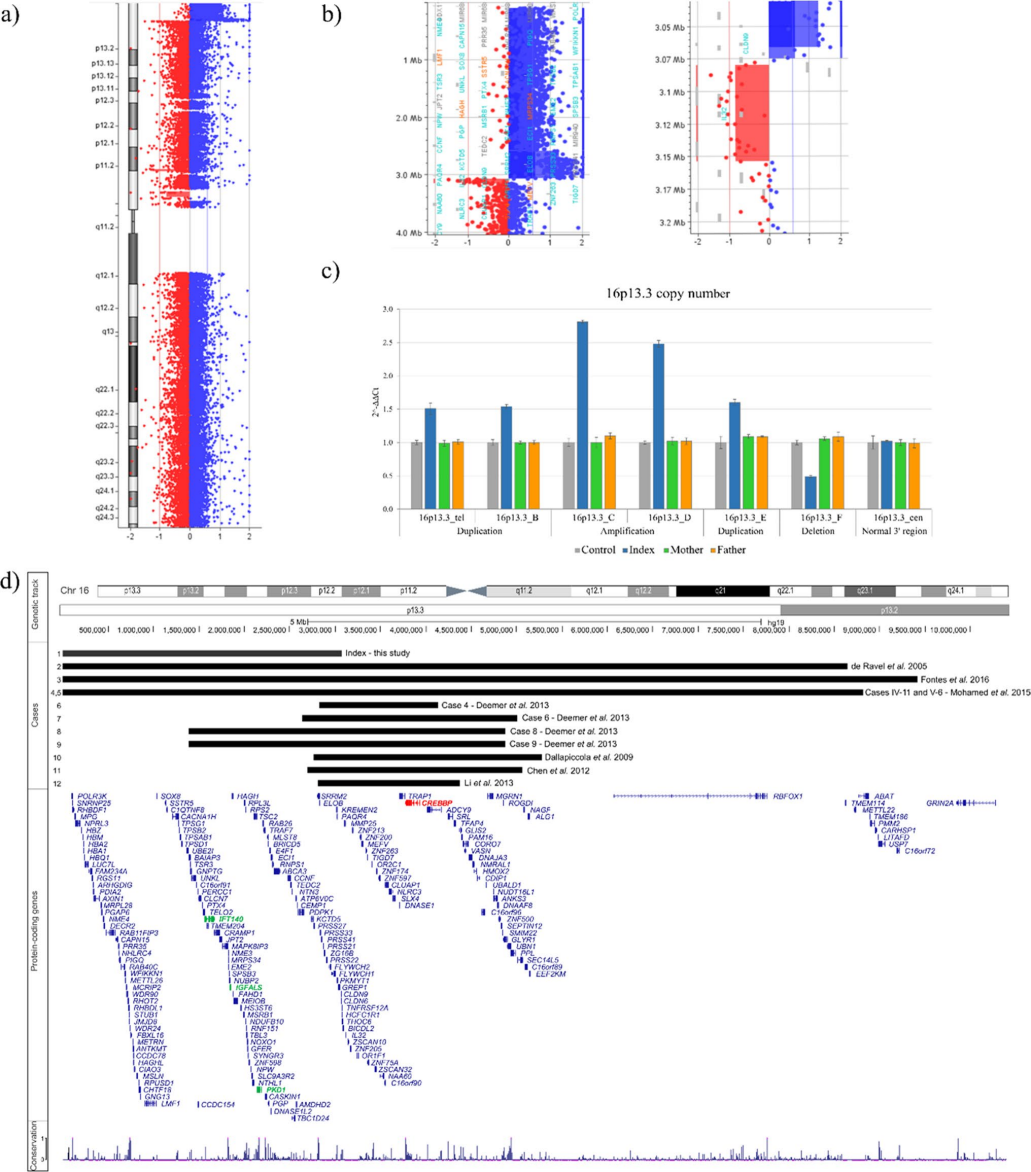
Results of aCGH and co-segregation analysis with an overview of the 16p13.3 genomic region. **a** An aCGH result overview of chromosome 16 performed in the index patient. **b** Zoom in on the detected aberrations: a 2.974 Mb duplication, an overlapping 477.448 kb amplification (left), and an adjacent deletion of ~ 74 kb (right). **c** qPCR results validating all SVs in the proband and excluding them in both unaffected parents, suggesting their de novo occurrence. **d** A comparison of the 16p13 aberrations detected in the proband (1) and eleven previously published cases (Chen et al. 2012; Dallapiccola et al. 2009; Demeer et al. 2013; Fontes et al. 2016; Li et al. 2013; Mohamed et al. 2015; de Ravel et al. 2005). Only four other individuals carried terminal SVs (cases 2–5). In all cases, except for the proband, the *CREBBP* gene was encompassed (depicted in red color). NCBI RefSeq Select gene track was used with one representative transcript per protein-coding gene—Annotation Release NCBI Homo sapiens 105.20220307 (12.03.2022) for the region chr16:1–10,394,218 (Kent et al. 2002), including *IFT140*, *IGFALS*, and *PKD1* (depicted in green color). The vertebrate Basewise Conservation by PhyloP was downloaded from UCSC (Kent et al. 2002)

Next, we performed a high-resolution array CGH in the index patient, which showed a previously unreported terminal duplication at chromosome 16p13.3, with the minimal size of 2.974 Mb (arr[GRCh37] 16p13.3(100359_3074565) × 3). Moreover, overlapping with the duplication, an amplification region was detected (arr[GRCh37] 16p13.3(2588831_3066278)amp), with a minimal size of 477.448 kb. A ~ 74 kb deletion was detected downstream of the aforementioned copy variants (Fig. 3a–b). qPCR analysis confirmed all SVs in the proband and excluded them in both unaffected parents, suggesting their de novo occurrence (Fig. 3c). The duplicated region encompassed 177 genes, including 135 protein coding, while the amplified fragment spans over 25 genes, of which 18 are protein-coding (Fig. 3d). The gene content of the aberrations and their overlap are shown in Table 1.

**Table 1:**
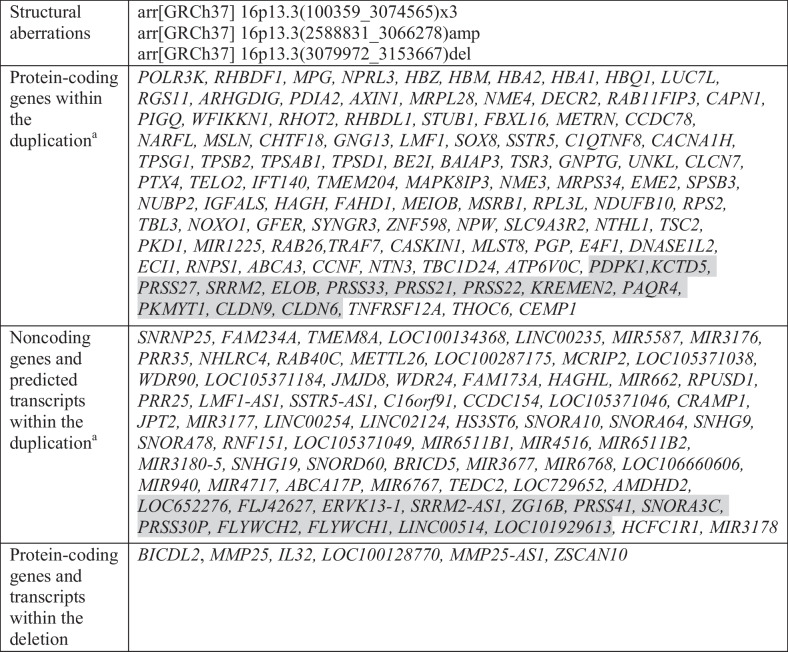
The gene content of the 16p13.3 aberrations and the overlap between identified duplication and amplification

^a^The amplified genes are highlighted in grey

## Discussion

In this report, we have described a female individual presenting with VSD, femoral hypoplasia with bowing, fractures of the femoral bones, umbilical hernia, micrognathia, cleft palate, low-set ears, and delayed psychomotor development. Based on the patient’s phenotype, especially facial dysmorphic features, we initially suspected DiGeorge syndrome or another 22q11.2 contiguous gene deletion disorder. Unexpectedly, array CGH identified a nearly 3 Mb terminal duplication of the short arm of chromosome 16. The molecular result pointed, therefore, to the partial 16p13.3 trisomy syndrome, with the clinical features of our index partially overlapping with the published patients harboring similar duplications. It is suggested that the minimal-causative region for the 16p partial distal trisomy encompasses the *CREBBP* gene (Demeer et al. 2013), which duplication is responsible for the most distinctive features of this syndrome, whereas the large size of the duplicated fragment, together with the type of the concomitant partial monosomy, are associated with limited life expectancy (Martin et al. 2002). In contrast to most reported cases, the duplicated region in the index does not involve the *CREBBP* gene and other neighboring genes. Nevertheless, the proband has a pattern of dysmorphic features characteristic of the described syndrome.

The proband described here carries a small deletion downstream of the duplicated region, while concurrent monosomy due to a reciprocal translocation is not observed; therefore, a pure trisomy 16p13.3 should be considered. The patient with pure trisomy 16p showing a similar phenotype to our proband was described by Martin et al. (2002). Interestingly, out of the most recognizable symptoms of this disorder, shortened long bones were observed in the affected individual, resembling our proband’s unique phenotype. However, several other features not noticeable in our proband, such as brain anomalies, pachygyria, optic nerve hypoplasia, genitourinary abnormalities, vesicoureteral reflux, and hydronephrosis, were observed. The variability of the clinical picture can be due to paternally derived balanced translocation observed in Martin’s patient and the difference in the gene content within duplications. Two other cases of a familial 16p terminal pure duplication were described by Cohen et al. (1983). Out of the most prominent signs of the syndrome, low birth weight, reduced occipital frontal circumference (OFC), apparently low-set ears, and psychomotor retardation were observed in the affected individuals. With the exception of the reduced OFC, these features are also present in our proband; however, the much bigger size of the duplicated segment encompassing *CREBBP* in the aforementioned cases (16p11- > p13) makes them only partially comparable to our index.

Other cases with terminal 16p duplication have also been reported in the literature. Among published cases, de Ravel et al. (2005), Mohamed et al. (2015), and Fontes et al. (2016) described the patients with 16p13.3 terminal duplication encompassing the *CREBBP* gene. Nevertheless, they presented similar clinical features to our patient, typically associated with the 16p13.3 duplication. The difference in phenotype between aforementioned patients and our index was probably due to the smaller size of the duplicated fragment and the absence of an associated translocation in our proband. In particular, our proband displayed multiple features common with the case of de Ravel, i.e., short femora, round face, hypertelorism, micrognathia, retrognathia, low-set ears, cleft palate, and psychomotor retardation (de Ravel et al. 2005; Thienpont et al. 2010). Unlike our proband, de Ravel’s patient carried de novo insertional translocation of chromosome 16p13.3 into the short arm of chromosome 22. While most of the clinical symptoms observed in our proband are highly unspecific and frequently occur in numerous chromosomal microaberrations, the shortening and hypoplasia of the femoral bones seem to be a rare congenital anomaly. Interestingly, femoral hypoplasia was also observed in another patient with 16p duplication (Rochat et al. 2007). In contrast to a small pure microaberration observed in our proband, the patient described by Rochat carried a duplication of almost the entire 16p region and a partial deletion of 2q, resulting from reciprocal parental translocation t(2;16)(q36;p11). Therefore, the genetic abnormalities in these two cases are not comparable.

Hypoplasia of the femoral bones represents a rare skeletal phenotype, with only few causative chromosomal aberrations described in the literature. First, Spielmann et al. (2016) showed that bilateral femoral hypoplasia in combination with unusual facies phenotype may result in a tiny interstitial microduplication of the 2q37.2 segment. The 1.9 Mb duplication is of a size similar to the CNV identified in our proband. Moreover, our recent study showed that isolated femoral bone hypoplasia can be caused by the duplications of around 0.5 Mb at the *FGF8 locus*, a key regulator of limb development (Socha et al. 2021). Both studies showed that chromosomal microaberrations might contribute to the development of the femoral hypoplasia phenotype. In order to unravel the possible link between hypoplastic femora and the microaberrations observed in our proband, we conducted a detailed analysis of the function of the genes involved in the reported region (Table 1). Out of the 177 genes located within the duplication region, *IGFALS*, *IFT140*, and *PKD1* are expressed in the femur and associated with bone development. *IGFALS* encodes acid-labile subunit (ALS), stabilizing IGF-I-IGFBP-3/5 binary complexes in serum (Kennedy et al. 2014). Insulin-like growth factor 1 (IGF-I), when bound to insulin-like growth factor-binding protein (IGFBP), plays an essential role in skeletal development and is a crucial mediator of bone mass during growth (Courtland et al. 2010). Courtland et al. studied the skeletal phenotype in ALS knockout (*ALSKO*) mice, which had reduced serum IGF-I levels. *ALSKO* mice displayed significantly smaller bone sizes and increased femur slenderness compared with their wild-type counterparts (Courtland et al. 2010). These results are in line with studies of humans with *IGFALS* mutations, which resulted in low bone mineral density and growth retardation (Domené et al. 2009). Another gene associated with femur development is *IFT140*. Zhang et al., using PCR-based methods, confirmed high *Ift140* expression in bones during their development. Furthermore, the expression of this gene was downregulated in long bones in an osteoporosis mouse model (Zhang et al. 2019). In another study, the deletion of *Ift140* in mice resulted in dwarfism, including reduced bone mass and shortened femoral length (Tao et al. 2019). Both papers suggest an essential role of *Ift140* in bone development. Another candidate gene is *PKD1*, which loss of function led to impaired bone development in mouse models. The study showed that the femur length in PKD1-deficient mice was shorter than that of control mice (Li et al. 2017). The results mentioned above indicated that *IGFALS*, *IFT140*, and *PKD1* play a significant role in the growth of femoral bones, when mutated contribute to abnormalities of the femur. Thus, the abovementioned genes can be linked to the skeletal phenotype of our proband; however, the mechanism through which an increased copy number of these genes leads to the bone malformation remains unknown. Functional studies unraveling the possible link between abnormalities of the femur and misexpression of any of the genes involved in the microduplication were not performed. Importantly, within the adjacent region of ~ 74 kb deletion identified in the proband, we were unable to find any candidate genes possibly involved in the skeletal development. Occurrence of lower limb malformations as a clinical symptom of 16p13 duplication syndrome have been also reported by Leonard et al. (1992), Mohamed et al. (2015), and Duarte-Bueno et al. (2020). The pattern of malformations and anomalies found in described individuals was similar to our proband’s clinical features. The studies conducted by Mohamed et al. (2015) and Duarte-Bueno et al. (2020) should be highlighted because the authors clearly showed an association between 16p13.3 microduplication and the skeletal phenotype. However, since the authors observed larger duplications of the 16p region, the possible genotype–phenotype correlations could not be clearly established.

Our proband presents with heart abnormality, which is rather a common feature of patients with 16p13.3 duplications (Chen et al. 2012; Demeer et al. 2013; Duarte-Bueno et al. 2020; Thienpont et al. 2007). In our index, as well as 35 other patients reported in the literature, congenital heart defects included 11/36 atrial septal defect (ASD), 8/36 VSD, and 4/36 valvulopathy (Duarte-Bueno et al. 2020). We analyzed all genes involved in the duplicated interval and pointed to the possible role of *NPRL3* and *IFT140*, as they are both expressed in the heart. In mouse models, the disruption of *Nprl3* resulted in cardiovascular defects, including VSD and led to embryonic death (Kowalczyk et al. 2012), while the mutant allele of *Ift140* was related to acute heart phenotype in embryos (Miller et al. 2013). Another study describing patients with an atrioventricular septal defect showed compound heterozygous variation in *IFT140* (Priest et al. 2016). The presence of heart malformations in mouse models for this locus confirmed its association with human cardiac defects. Therefore, the above-described genes could be evaluated when searching for candidate genes contributing to heart abnormalities in patients with partial trisomy 16p syndrome. The analysis pointed to *IFT140*, suggesting that the mutation of this gene in our proband might lead simultaneously to heart abnormalities and hypoplastic femora. Moreover, *NPRL3* and *IFT140* are located in the microduplication region, similarly to genes that might contribute to the femoral phenotype (*IGFALS*, *IFT140*, *PKD1*).

In conclusion, our article reports a novel 16p13.3 duplication of ~ 3.0 Mb in a patient with distinct clinical features (i.e., bilateral shortening and fractures of the femoral bones and shortened humeral segments), substantiating and expanding the phenotypic spectrum correlated with this syndrome. We pointed to the *IGFALS*, *IFT140*, and *PKD1* genes localized in the microduplication detected in our proband which code essential proteins for long bone development. We assume that the gene dosage effect of the abovementioned genes can be related to the skeletal phenotype observed in our patient. To the best of our knowledge, this is the first reported case with 16p13.3 terminal duplication of the size below 3 Mb. Moreover, the duplicated region of our proband does not encompass *CREBBP*, which makes our index unique. Therefore, our proband with her detailed phenotypic description may be helpful for clinicians who consult patients with this syndrome. Further studies such as breakpoint analysis of pathogenic 16p13 CNVs are needed to shed more light on the genotype–phenotype correlations.

## Supplementary Information

Below is the link to the electronic supplementary material.

Supplementary file1 (DOCX 17.9 KB)

## Data Availability

All data generated or analyzed during this study are included in this article. Phenotypic data along with the array CGH results were submitted to the DECIPHER database (http://decipher.sanger.ac.uk); accession number is 490639.
