# Supplementary material for: A pure de novo 16p13.3 duplication and amplification in a patient with femoral hypoplasia, psychomotor retardation, heart defect, and facial dysmorphism—a case report and literature review of the partial 16p13.3 trisomy syndrome
Source: J Appl Genet. 2022 Dec 31;64(1):125–34. doi: 10.1007/s13353-022-00743-7 (PMC9837002; doi:10.1007/s13353-022-00743-7)
Supplement: Supplementary file 1 — Supplementary file1 (DOCX 17.9 KB) [file 13353_2022_743_MOESM1_ESM.docx]

A pure *de novo* 16p13.3 duplication and amplification in a patient with femoral hypoplasia, psychomotor retardation, heart defect and facial dysmorphism – a case report and literature review of the partial 16p trisomy syndrome

Journal of Applied Genetics

Magdalena Socha,^1^ Anna Szoszkiewicz^1^, Dorota Simon,^2^ and Aleksander Jamsheer,^1,2^*

^1^Department of Medical Genetics, Poznan University of Medical Sciences, Rokietnicka 8, 60-806 Poznan, Poland

^2^Centers for Medical Genetics GENESIS, Dąbrowskiego 77A, 60-529 Poznan, Poland

*Corresponding author:

Aleksander Jamsheer, MD, PhD, MSc

E-mail: [jamsheer@wp.pl](mailto:jamsheer@wp.pl)

Supplementary Information

Table S1 Oligonucleotide primers used for validation and co-segregation analyses of the CNVs

| Primer | Sequence 5’ 🡪 3’(Hg19) | Genomic coordinates | Target |
| --- | --- | --- | --- |
| ALB_conF | TGAAATGGCTGACTGCTGTG | chr4:74274367-74274449 | Autosomal reference |
| ALB_conR | GGAGGTTTGGGTTGTCATCT |  |  |
| F8_conF | TTTCCATTCAACACCTCAGTCGT | chrX:154227766-154227850 | X chromosome reference |
| F8_conR | GCCTTGGCTTAGCGATGTTG |  |  |
| q16p13.3_telF | TCCCCCTCGAGAATACACAC | chr16:84273-84354 | Duplicated region |
| q16p13.3_telR | ACCTGCTGAGTGTGCTTGTG |  |  |
| q16p13.3_BF | TGGTTGGGTTTCAAATGGTT | chr16:1033030-1033113 |  |
| q16p13.3_BR | AGCTCCAAGCTCCCTTCTTC |  |  |
| q16p13.3_CF | TTGTGGGGAATTTGCTTAGG | chr16:2809414-2809497 | Amplified region |
| q16p13.3_CR | CCCTTTACCAGTCAGGGACA |  |  |
| q16p13.3_DF | TGCACAATCTGAGCCAAGTC | chr16:3036404-3036486 |  |
| q16p13.3_DR | GCTACTGGGAGGAACTGCTG |  |  |
| q16p13.3_EF | TGCAGACAGAGGTGTTCCAG | chr16:3075704-3075787 | Duplicated region |
| q16p13.3_ER | CCAGAAACTTCCCACATGGT |  |  |
| q16p13.3_FF | GTGGCTTTAGGGTCATGCTC | chr16:3088029-3088111 | Deleted region |
| q16p13.3_FR | TGGGCAAGTCACTCTCACTG |  |  |
| q16p13.3_cenF | TGTTGCCTAGGGATGCTCTT | chr16:3155707-3155786 | Normal 3’ region |
| q16p13.3_cenR | GCCTCTCACAGTTGGGAAAA |  |  |
